# Urinary Deposits and Treatment

**Published:** 1845-10

**Authors:** Golding Bird


*Urinary Deposits and Treatment.* By Golding Bird, A.M., M.D.

*Urine without any visible Deposit.*—A piece of litmus paper should be immersed in the urine, which, if acid, will change the blue color of the paper to red. Should no change occur, a piece of reddened litmus paper must be dipped in, and if the secretion be alkaline, its blue color will be restored; but if no change occur, the urine is neutral. Some of the urine should then be gently heated in a polished metallic spoon over a candle, or, what is preferable, in a test-tube over a spirit-lamp, and if a white deposit occurs, albumen or earthy phosphates are present; the former if a drop of nitric acid does not re-dissolve the deposit, the latter if it does. If the urine be very highly colored, and undergoes no change by boiling, the coloring matters of bile, *blood*, or purpurine are present. To determine which, pour a thin layer of urine on the back of a white plate, and allow a few drops of nitric acid to fall in the centre; an immediate and rapidly ending play of colors, from green to red, will occur if bile, but no such change if purpurine alone exists. Should the highly-colored urine alter in color or transparency by heat, the presence of blood must be suspected.

If the addition of nitric acid to deep red urine, unaffected by heat, produces a brown deposit, an excess of uric acid exists. If the urine be pale, immerse the gravimeter, and if the specific gravity be below 1·012, an excess of water exists in the urine, but if above 1·025, the presence of sugar, or excess of urea is indicated. To determine which, place a few drops in a watch-glass, add an equal quantity of nitric acid, and allow the glass to float on some cold water; crystallization of nitrate of urea will occur in two or three minutes, if the latter exists in excess. Should this change not occur, the urine must be examined specially for sugar, which, it must be remembered, may exist in small quantities, without raising the specific gravity of the fluid.

Should the urine be alkaline, add a drop of nitric acid; if a white deposit occurs, albumen is present; if brisk effervescence follows the addition of the acid, the urea has been converted into carbonate of ammonia.

*Urine depositing a visible sediment.*—If the deposit is flocculent, easily diffused on agitation, and scanty, not disappearing on the addition of nitric acid, it is chiefly made up of healthy mucus, epithelium, or in a woman, admixture of leucorrhœal discharge. If the deposit is ropy and apparently viscid, add a drop of nitric acid; if it wholly or partially dissolves, it is composed of phosphates, if but slightly affected, of mucus. If the deposit falls like a creamy layer to the bottom of the vessel, the supernatant urine being coagulable by heat, it consists of pus. If the deposit is white, it consists of urate of ammonia, phosphates, or cystine; the first disappears on heating the urine, the second on the addition of a drop of diluted nitric acid, whilst the third dissolves in ammonia, and the urine generally evolves an odor of sweet-briar. If the deposit be colored, it consists of red particles of blood, uric acid, or urate of ammonia, stained with purpurine. If the first, the urine becomes opake by heat; if the second, the deposit is in visible crystals; if the third, the deposit is amorphous, and dissolves on heating the fluid.

Oxalate of lime is often present diffused through urine, without forming a visible deposit; if this be suspected, a drop of the urine examined microscopically will detect the characteristic crystals. Much of the little time required for the investigation thus sketched out, may be saved by remembering the following facts:—

If the deposite be white, and the urine acid, it in the great majority of cases consists of urate of ammonia; but should it not disappear by heat, it is phosphatic. If a deposit be of any color inclining to yellow, drab, pink, or red, it is almost sure to be urate of ammonia, unless visibly crystalline, in which case it consists of uric acid.

The only apparatus and re-agents required for these investigations at the bed-side are—A gravimeter, made small enough to float in an ounce of fluid. Red and blue litmus paper. A test-tube and watch-glass. Nitric acid. All these are readily arranged in a little case, and can thus be always at the convenience of the practitioner. For the microscopic examination of the urine, a vertical instrument on a firm tripod stand, and large ring-stage, provided with a good half-inch achromatic object-glass, is alone required.

The following table briefly points out the best mode for the analytical examination of saline deposits, either by chemical tests or the microscope. The latter mode of investigation is infinitely preferable to all others, both for accuracy and economy of time, but is of course not readily available in the sick-room.

*A Table for discovering the nature of saline deposits by chemical re-agents.*

| | | | |
|---|---|---|---|
| 1. | Deposit, | white - - - - - | 2. |
| | —— | colored - - - - - | 5. |
| 2. | —— | dissolves by heat - - | Urate of ammonia. |
| | —— | insoluble by heat - - | 3. |
| 3. | —— | soluble in liquor ammoniæ | Cystine. |
| | —— | insoluble in - - - - | 4. |
| 4. | —— | soluble in acetic acid - | Earthy phoshates. |
| | —— | insoluble - - - - - | Oxalate of lime. |
| 5. | —— | visibly crystalline - - | Uric acid. |
| | —— | amorphous - - - - | 6. |
| 6. | —— | readily soluble by heat - | Urates. |
| | —— | slowly dissolves by heat - | —— stained by purpurine. |

*Table for determining the nature of saline deposits by the microscope.*

| | | | |
|---|---|---|---|
| 1. | Deposit, | white - - - - - - | 2. |
| | —— | colored - - - - - | 5. |
| 2. | —— | an amorphous powder - | Insoluble by heat; Phosphate of lime. Soluble by heat; Urate of Am. |
| | —— | in defined crystals - - | 3. |
| 3. | —— | in prismatic crystals - - | Triple phosphate. |
| | —— | in octohedral or tabular crystals | 4. |
| 4. | —— | in octohedra - - - - - | Oxalate of lime. |
| | —— | in simple or compound tables | Cystine. |
| 5. | —— | in transparent crystals - | Uric acid. |
| | —— | amorphous, or spherical masses | Urates ammonia or soda. |

There is one error in these directions, pointed out by Dr. James Johnson, viz: where Dr. Bird says that "if the urine be very highly colored, and undergoes no change by boiling, the coloring matters of bile, *blood*, or purpurine are present;" for it is well known that both the color and transparency of bloody urine are changed by heat. Dr. Johnson also points out that the headings of these tables are not quite correct, as neither uric acid nor cystine can strictly be called "saline" substances. On the Medical Treatment of the different forms of Uric Acid Gravel, Dr. Bird observes, that

Discarding altogether the existence of any specific agent for a disease which is rather symptomatic of another affection than really idiopathic, the therapeutical agents may be briefly referred to the following heads.

1. *Attention to the function of the skin.*—The remarks already made on the effect of an arrest of perspiration furnishing a pabulum for the formation of a deposit, or by retaining in the circulation a substance capable of rendering uric acid insoluble, show the necessity of attending to this indication. I have repeatedly seen diaphoretics, warm clothing, the use of a flannel, and in winter, even a chamoise-leather waistcoat, with friction by means of a flesh-brush or hair-glove, repeatedly remove a deposit of uric acid gravel, and in more than one instance, where even an hereditary taint existed from gouty or calculous progenitors. My own experience induces me to regard the warm, or still better, the vapor bath, as the most valuable diaphoretic. The latter is readily employed in private practice by means of the very convenient and portable apparatus of M. Duval, which has for a long time superseded other forms of vapor bath at Guy's Hospital. Actual diaphoresis is by no means necessary in the treatment of all cases of uric gravel; friction to the skin, and, when persons are sufficiently robust, immersion in the cold bath, followed by rubbing the surface of the body with a dry and rough towel, until reaction is produced, is often of great service.

*Restoring the tone of the organs of digestion.*—By effecting this, a double object is attained; the perfection of the primary assimilation of the food by which the entrance of crude nitrogenised matter, capable of being converted into uric acid, into the blood is prevented, and the prevention of the generation of any acid, the product of unhealthy digestion, which might be absorbed by the lacteals, and act as a precipitant of uric acid. This part of the treatment of calculous affections must be modified by the peculiarities of the case, and indeed is identical with that of the different forms of dyspepsia. Careful attention to the bowels, avoiding excessive purging, the use of minute doses of mercury, as of a grain of pil. hydrargyri or hydrarg. cum creta, with thrice that quantity of ext. conii, administered two or three times a day, with moderate doses of the carbonate of potassa or soda in the mist. gentianæ comp., if constipation exists, or in inf. calumbæ, or what is far better, from its action on the skin, inf. serpentariæ, will often effect immense relief. Where gastrodynia, with or without pyrosis, exists, the use of a half a grain of argenti nitras, or one of argenti oxydum, immediately before a meal, will often check alike the gastric and renal symptoms. But the most important element in the treatment is a rigid attention to the quality and quantity of the ingesta, taking the utmost care to select those articles of diet which the patient can best digest, it being of far greater importance, in the majority of cases, to regard this, than to choose articles of food according to their chemical composition. A too bulky meal of animal or vegetable food is injurious to persons laboring under calculous dyspepsia, for whilst the former supplies too much nitrogen, both will become sources of mischief by overloading the digestive functions, and preventing the chylopoietic viscera doing their duty. In protracted cases, however, much good is derived by actually cutting off part of the supply of nitrogen. In this way I have seen a copious deposit of uric acid gravel disappear, after other measures had failed to give relief.

Moderate muscular exertion, and a due amount of exercise is quite essential in the treatment of this disease; for not only do they call into play some very important functions, but often improve the general health. Besides this, when the stomach is imperfectly able to digest nitrogenised food, exercise will often aid its assimilation by making a call upon the chylopoietic organs for supply for the want of tissue it produces. Among the remedies which appear most successful when the food is not converted into healthy chyle, and an unhealthy state of the blood from the presence of imperfectly assimilated matters results, the preparations of iron deserve notice. I have repeatedly seen copious deposits of uric acid in persons of low power completely disappear *pari passu* with the cure of the pseudo-chlorotic symptoms present, by the use of this important drug. The best mode of administering it, is in combination with a vegetable acid, as the stomach bears it well in this form, and it is probably more likely to enter the circulation. From six to twelve grains of the ammonio-citrate, or ammonio-tartrate of iron taken thrice a day immediately after a meal in a glass of water, have been most successful. The solution of the sesqui-acetate of iron is also a very valuable preparation, but is often inconvenient to prescribe, in consequence of its not being of constant strength.

Dr. Bird then proceeds to notice remedies which act as solvents of uric acid: and successively passes under review alkalies and their carbonates, biborate and phosphate of soda, benzoic and cannamic acid.

As the alkaline urates are far more soluble than the free acid, the employment of soda and potass with their carbonates has been long used in the treatment of uric gravel. They moreover exert a beneficial effect in neutralizing any free acid in the primæ viæ, and thus preventing a precipitant of uric acid reaching the kidneys. The liquor potassæ may be employed in doses of half a drachm thrice a day: it is best taken about half an hour after a meal, and may be conveniently administered in a little bitter ale, which conceals much of its disagreeable flavor, or in any bland vehicle. The carbonate of potass and soda are, however, far more agreeable, and perhaps more efficient remedies—of these the bicarbonate of potass deserves the preference. It should be given thrice a day in doses of ℈j. or ʒss. I think it appears to act best when in a glass of warm water. To make it more agreeable, I generally order, what I am accustomed to term to my patients, the artificial Vichy water, made by stirring ʒss. of bicarbonate of potass and gr. v. citric acid into a tumbler of lukewarm warm. This mixture evolves enough carbonic acid to be 'sparkling,' and is generally taken with readiness.

A very convenient mode of impregnating the urine with an alkali is to administer the potass or soda in combination with a vegetable acid, especially with the acetic, citric, or tartaric. The mode in which these act is easily explained; when acetate, citrate, or tartrate of potass are ignited, the acid absorbs oxygen, and is converted into carbonic acid and water, part of the former uniting with the alkali. In a similar manner are the salts decomposed during the process of healthy digestion; a carbonate finds its way into the circulation, and reaching the kidneys, renders the urine alkaline. If, however, the digestive powers are impaired, the vegatable acid is only partly decomposed, and in some few persons it escapes the influence of digestion altogether. 114 grains of tartrate of potass, 106 of citrate, and 99 of the acetate, absorb respectively 40, 48, and 64 grains of oxygen, to be converted into carbonate of potass and water. These salts may be administered by directing the use of the common saline powders made with carbonates of potass or soda and the citric or tartaric acid, in effervescence. When not contra-indicated the use of strawberries, currants, and some other fruits containing alkaline citrates and malates, are capable of making the urine alkaline, and may be occasionally employed with advantage.

Some persons cannot bear the use of free or carbonated alkalies without suffering severely in their general health, nor is their protracted use altogether without some ill effect. A flabby state of the muscles, and an emaciated condition of the system, is frequently produced by the persistent use of alkaline remedies. Their injudicious employment may, as Dr. Prout has suggested, produce the formation of oxalic acid. Uric acid is soluble in a solution of borax, the biborate of soda—more so, indeed, than in alkaline carbonates; and this salt may be taken for some time, at least by male patients, without producing any very injurious constitutional effects, and readily finds its way into the urine. On this account its administration has been suggested in cases of uric acid gravel, but it has not been much employed in this country. In women, this drug cannot be employed with impunity, as it certainly exerts a stimulant action on the uterus, and I have seen it in two instances produce abortion.

The remarkable solvent action of phosphate of soda on uric acid, to which Liebig has lately directed attention, inspires a hope that its administration might be of use in cases of calculous disease, by impregnating the urine with an active solvent. All that is required to insure this drug reaching the urine is to administer it in solution sufficiently diluted; ℈j. to ʒss. might be administered in any vehicle, as in broth or gruel, as when diluted, the phosphate tastes like common salt, and few persons object to its flavor. I have administered this drug in two very chronic cases of uric acid gravel, and in one with the effect of rapidly causing a disappearance of the deposit. This occurred in the person of a lady about forty years of age, who had, at my wish, for some weeks used the artificial Vichy water of the German Spa at Brighton without relief. The triple salt, ammonia-phosphate of soda, would perhaps be a more active remedy than the simple phosphate, but its disagreeable flavor constitutes one objection to its employment.

Much attention has been lately drawn to the effects of benzoic acid in preventing the formation of uric acid, by the observations of Mr. Alexander Ure. When this acid or its salts are administered, they are acted upon by the stomach in a very different manner from the other vegetable acids. Instead of becoming oxidized, and being converted into carbonic acid, it combines with those nitrogenised elements which would otherwise have formed urea or uric acid, and is converted into hippuric acid. It has been stated that the quantity of uric acid falls, when the benzoic acid is administered, below the average quantity, or even disappears from the urine. This has been, however, shown by Dr. Garrod, to be an error, and that urea alone disappears. Be this as it may, it is certain that the acid does appropriate to itself some body rich in nitrogen to form hippuric acid; and experience has shown that, in cases where an excess of uric acid is secreted, the admistration of this drug appears to limit it to about the normal quantity.

*Directions for detecting the existence of the oxalate of lime deposits.*—To examine urine for the purpose of detecting the existence of the salt under consideration, allow a portion passed a few hours after a meal to repose in a glass vessel. Decant the upper 6-7ths of the urine, pour a portion of the remainder into a watch-glass, and gently warm it over a lamp; in a few seconds the heat will have rendered the fluid specifically lighter, and induced the deposition of the crystal of oxalate, if any were present; this may be hastened by gently moving the glass, so as to give the fluid a rotary motion, which will collect the oxalate at the bottom of the capsule. The application of warmth serves, also, to remove the obscurity arising from the presence of urate of ammonia, which is readily dissolved by exposing urine containing it, to a gentle heat. Having allowed the urine to repose for a minute or two, remove the greater portion of the fluid with a pipette, and replace it by distilled water. A white powder, often of a glistening appearance, will now become visible, and this, under a low magnifying power, as by placing the capsule under a microscope furnished with a half inch object-glass, will be found to consist of crystals of oxalate of lime in beautifully formed transparent octohedra, with sharply defined edges and angles.

The crystals of the oxalate, when collected in the manner above directed in a watch-glass, are unaltered by boiling either in acetic acid or solution of potass. In nitric acid they readily dissolve without effervescence. The solution may be very readily watched under the microscope. When the oxalate is allowed to dry on a plate of glass, and then examined, each crystal presents a very curious appearance, resembling two concentric cubes, with their angles and sides opposed, the inner transparent, and the outer black, so that each resembles a translucent cube set in a black frame. This is best observed under a half inch object-glass; as with a higher power this appearance is lost.

In a very few cases the oxalate is met with in very remarkable crystals, shaped like dumb-bells, or rather like two kidneys with their concavities opposed, and sometimes so closely approximating as to appear circular, the surfaces being finely striated. These crystals are produced, in all probability, by a prolific arrangement of minute acicular crystals.

*On the Treatment of Oxaluria.*—The treatment, in the majority of cases, is very successful; a few only resisting all the plans which were adopted. As a general rule, the functions of the body, where obviously imperfect, should be corrected, the general health attended to by the removal of all unnaturally exciting or depressing influences, the skin should be protected from sudden alterations of temperature by a flannel or woolen covering, and the diet carefully regulated. This has generally consisted of well cooked, digestible food, obtained in about equal proportions from the animal and vegetable kingdom; all things which tend to produce flatulence being carefully avoided. The drink should consist of water or some bland fluid, beer and wine being excluded, especially the former, unless the patient's depression render such positively necessary. A very small quantity of brandy in a glass of water has generally appeared to be the most congenial beverage at the meals. The administration of nitric acid, as suggested by Dr. Prout; or, what appeared to be preferable, the nitro-hydrochloric acid, in small doses, in some bitter infusion, or laxative mixture, as the mistura gentianæ comp., was, with minute doses of mercury, generally successful, if continued a sufficient length of time. In cases where these failed, active tonics, especially the sulphate of zinc, and where the patient was emaciated or chlorotic, the salts of iron in very large doses, appeared to be of great use, by subduing the irritable state of the nervous system. The shower bath by acting in a similar manner, has been also of great service. There is one remedy which appears to exercise a marked influence over the characters of the urine, and which, from the small amount of experience I have had with it, seems to hold out the probability of its great utility in the disease under consideration: I allude to the colchicum, which, it is now generally admitted, exerts an immense influence over the organic system of nerves, and the functions under its control. The character of the urine is remarkably influenced by this drug, an excess of uric acid generally being present during its administration; and in two cases in which oxalate of lime existed in abundance before its employment, uric acid appeared after a few days as a deposit, and nearly entirely replaced the oxalate; a circumstance generally observed during the successful treatment of this disease by other remedies. In no case have I seen the disease suddenly yield; it has generally slowly disappeared *pari passu* with the decrease in number and size of the crystals of the oxalate.

*On the Pathological Indications of Phosphates.*—The occurrence of deposits of the earthy phosphates in the urine, must be regarded as of serious importance, always indicating the existence of important functional, and too frequently, even of organic mischief. One general law appears to govern the pathological development of these deposits, viz., that they always exist simultaneously with a depressed state of nervous energy, often general, rarely more local, in its seat. Of the former, the result of wear and tear of body and mind in old people, and of the latter, effects of local injury to the spine, will serve as examples. It is true, that in the majority of these cases there is much irritability present, there is often an excited pulse, a tongue white on the surface, and red at the margin and tip, with a dry, often imperspirable, occasionally hot skin. Still it is irritability with depression, a kind of erythism of the nervous system, if the expression be permitted, like that observed after considerable losses of blood.

Dr. B. recognizes at least four different pathological conditions connected with deposits of earthy phosphates.

1. Cases in which dyspepsia, with some febrile and nervous irritation, exists, independently of any evidence of antecedent injury to the spine.
2. Cases characterised by high nervous irritability, with a varying amount of marasmus, following a blow or other violence inflicted on the spine, but without paralysis.
3. Cases in which the phosphatic urine co-exists with paraplegia, the results of spinal lesion.
4. Cases of diseased mucous membrane of the bladder.

The treatment of the first class of cases must be rather directed by general principles than limited to the solution of the phosphatic deposits.

It is true that by the persistent administration of acids the deposits may disappear for a time, but the ailment goes on; all that is effected by such treatment is to mask a symptom, and an important one, of the progress of the malady. After having attended to the morale of the case, as far as possible rousing the patient from any morbid influence excited in his mind, whether real or imaginary, the next thing is to attend to the general health. The bowels should be freed from any unhealthy accumulation by a mild mercurial laxative, as a few grains of pil. hydrarg., followed by a dose of rhubarb or castor oil; but all active purging should be avoided, as it generally aggravates the distress of the patient, and decidedly interferes with the success of the treatment. A combination of a tonic laxative with a sedative may then be administered, as tinct. hyoscyami et sp. ammon. aromatici ää *m*xx—ʒss. ex mist. gentianæ co. ℥j. ter in die. If the bowels be irritable, the inf. cascarillæ, or inf. serpentariæ, may be substituted for the mist. gentianæ comp. Should gastrodynia exist, great relief will be obtained by the administration of half a grain of oxide of silver, made into a pill with confection of opium, before a meal. The diet should be very carefully regulated, all bland nutritious articles of food being preferred; vegetables should be avoided, and in general a small quantity of good sherry may be allowed. By a plan of treatment of this kind, the patients generally do well, and the phosphates and excess of urea vanish from the urine. As the patient approaches convalescence, much good is often effected by the use of the sulphate of zinc in gradually increasing doses, beginning with a grain thrice a day, made into a pill with a little ext. hyoscyami, or ext. gentianæ, and increasing the quantity every three or four days, until five grains or more are taken at a dose. Under the use of the zinc, I have seen many cases do well, whose symptoms approached in severity and character those of the mild delirium tremens. I need hardly say that change of scene and occupation are important adjuvants to our medical treatment.

The second class of cases are far less amenable to treatment.

In these, the phosphatic deposit is often copious, and sometimes consists nearly exclusively of phosphate of lime; the lumbar pain and weight are considerable, the skin often dry and scarcely perspirable; in some cases, indeed, I have seen it look as if varnished; the tongue sometimes white, is often red; the thirst often great; indeed, the general appearance of the case closely resembles one of diabetes. The urine is generally more copious than natural, frequently pale, and of a specific gravity below the average. On investigating the patient's history, some evidence of a previous strain or wrench of the back, or a blow over the spine, is always elicited. These patients are seldom hypochondriacal; but intense irritability of temper, and a painful, anxious expression of countenance and manner are almost inevitably present.

In the treatment of these cases, the great end and aim must be to subdue the morbidly irritable state of the brain and nervous system; and, subsequently, by a generous diet and persistent use of those tonics which appear especially to exert their influence on the organic nerves, as silver, bismuth, zinc, &c., to endeavor to restore the assimilative functions to their due vigor. Besides the general indications to be fulfiled by regulated diet, amusements, exercise, &c., the use of narcotics, especially of opium, or the preparations of morphia, should be regarded as of the highest value; and we are indebted to Dr. Prout for first directing the attention of the profession to their use.

Cases occasionally appear in which the symptoms are of a much milder character, but which insidiously go on to the formation of a calculus. It is in these in particular that the use of acids is called for, to hold the phosphatic salts in solution, and prevent their being moulded into a concretion in the pelvis of the kidney. Unfortunately there is a great uncertainty attending their use; sometimes the mineral acids appear to reach the urine and destroy its alkaline character; often however, even their continued employment appears to be utterly ineffectual in rendering the urine acid. So far as I have watched cases of this kind, the nitric acid has appeared to produce the smallest amount of gastric derangement, and to render the urine acid, or at least diminish its alkaline reaction. In one case lately, in which the nitric acid could not be borne, the phosphoric appeared to succeed.

In the third class of cases, the deposition of the phosphates is a sure symptom of a grave and serious lesion, and must be treated according to the particular disease existing. The fourth class of cases, or "those in which the phosphates are probably entirely secreted with unhealthy mucus by a diseased lining membrane of the bladder," are familiar to every practitioner.

One point of great practical consequence must be borne in mind in forming a prognosis from the state of the urine, viz: not to regard it as ammoniacal, because the odor is offensive; and not to consider the deposit as purulent because it looks so. A piece of litmus paper will often show the urine to be really acid, and microscopic inspection often proves that the puriform appearance of the urine is owing to the abundance of phosphates with mucus. For want of these precautions I have seen one or two cases regarded as almost hopeless, which afterwards yielded to judicious treatment. It is quite certain that the mucous membrane of the bladder may, under the influence of chronic inflammation, secrete so much of the earthy phosphates and unhealthy mucus as to render the urine puriform and offensive without having necessarily undergone any structural change. A few cases have occurred to me in practice, in which the kind of urine just referred to was secreted for a long time, and yet yielded readily to treatment. In these, the greatest good has arisen from freeing the bladder from the phosphates which appear almost to incrust it, by acid injections. In this way cases have occasionally yielded which have quite defied all other treatment.

In one case which Dr. Bird relates, great benefit was obtained by the daily careful injection of acid. hydrochlor. *m*x, with vin opii *m*xx. in barley water. With respect to the influence of diuretics, Dr. Bird lays down three 'general laws' which regulate the action of these remedies.

*Law 1st.* All therapeutical agents intended to reach the kidneys must either be in solution when administered, or capable of being dissolved in the fluids contained in the stomach or small intestines, after being swallowed.

*Law 2d.* Bodies intended to reach the kidneys must, to insure their absorption, have their solutions so diluted as to be of considerably lower density than either the liquor sanguinis, or serum of blood.

*Law 3d.* If a sufficient quantity of water cannot be received into the small intestines, or the circuit through the portal system in the vena-cava ascendens, or thence through the lungs and heart into the systemic circulation, be obstructed, or if there be extensive disorganization of the kidneys, the due secretions cannot be effected.

1. Whenever it is desirable to impregnate the urine with a salt, or to excite diuresis by saline combination, it must be exhibited in solution, so diluted as to contain less than five per cent. of the remedy, or not more than about twenty-five grains in an ordinary draught. The absorption of the drug into the capillaries will be insured by a copious draught of water, or any diluent, immediately after each dose.

2. When the urine contains purpurine, or other evidence of portal obstruction exist, the diuretics or other remedies employed should be preceded or accompanied by the administration of mild mercurials—taraxacum, hydrochlorate of ammonia, or other cholitic remedies. By these means, or by local depletion, the portal vessels will be unloaded, and a free passage obtained to the general circulation.

3. In cases of valvular obstructions existing in the heart and large vessels, it is next to useless to endeavor to excite diuretic action, or appeal to the kidneys by remedies intended to be excreted by them. The best diuretics here will be found in whatever tends to diminish the congested state of the vascular system, and to moderate the action of the heart; as digitalis, colchicum, and other sedatives, with mild mercurials.—*Medico-Chirurgical Review*, *April*, 1845.—*Ibid.*

